# Generalized Pustular Psoriasis and Systemic Organ Dysfunctions

**DOI:** 10.3390/ijms25116270

**Published:** 2024-06-06

**Authors:** Romane Teshima, Natsuko Saito-Sasaki, Yu Sawada

**Affiliations:** Department of Dermatology, University of Occupational and Environmental Health, Kitakyushu 807-8555, Japan

**Keywords:** generalized pustular psoriasis, systemic organ dysfunctions, IL-36

## Abstract

This review explores the intricate relationship between generalized pustular psoriasis (GPP) and various systemic diseases, shedding light on the broader impacts of this severe skin condition beyond its primary dermatological manifestations. GPP is identified as not only a profound contributor to skin pathology but also a significant risk factor for systemic diseases affecting cardiovascular, hepatic, renal, pulmonary, and skeletal systems, as well as associated with an increased incidence of anemia, depression, anxiety, and arthritis. The research highlights the complex interplay of cytokines, particularly IL-17 and IL-36, which are central to the pathophysiology of GPP and implicated in the exacerbation of systemic conditions. Key findings indicate a higher incidence of cardiovascular events in GPP patients compared to those with other severe forms of psoriasis, notably with a stronger correlation between myocardial infarction history and GPP development. Liver disturbances, frequently reversible upon psoriasis remission, suggest a cytokine-mediated link to hepatic health. Renal dysfunction appears elevated in GPP sufferers, with IL-17 and IL-36 potentially driving renal fibrosis. Similarly, interstitial lung disease and osteoporosis in GPP patients underscore the systemic reach of inflammatory processes initiated in the skin. The associations with anemia, depression, anxiety, and arthritis further complicate the clinical management of GPP, requiring a multidisciplinary approach. The study concludes that managing GPP effectively requires a holistic approach that addresses both the cutaneous and systemic dimensions of the disease, advocating for continued research into the mechanisms that connect GPP with broader health implications to refine therapeutic strategies.

## 1. Introduction

The skin, as the body’s outermost organ, serves a critical role in defending against a variety of external stimuli [1,2]. Beyond its barrier function, the skin is intricately involved in the pathogenesis of systemic inflammatory diseases [3,4,5]. Various dermatological conditions demonstrate how inflammation can originate from the skin and spread to other organs, thereby playing a crucial role in the development of systemic inflammatory diseases [6,7,8]. For example, psoriasis is associated with an increased risk of cardiovascular events and mental health disorders [9,10,11], highlighting the clinical necessity of managing skin inflammation to mitigate broader health risks in the future [3].

Generalized pustular psoriasis (GPP) is a severe inflammatory skin condition characterized by the rapid development of numerous painful pustules across the body, accompanied by systemic symptoms like high fever [12,13,14]. In extreme cases, GPP can escalate to critical conditions such as multi-organ failure [9]. This distinguishes GPP from more prevalent forms of psoriasis, such as plaque psoriasis, and emphasizes the critical need for effective management during the acute phase [15,16]. Studies have documented that the systemic inflammatory response initiated by GPP-related skin eruptions can affect various body organs significantly, indicating the potential for widespread organ involvement.

This study aims to provide a more comprehensive analysis of the systemic effects of inflammatory skin diseases on various organs, focusing primarily on GPP. Although research in this area remains limited, this report seeks to consolidate and discuss the broader systemic implications based on existing case studies and literature. This approach not only enhances our understanding of the pathophysiology of GPP but also guides future therapeutic strategies aimed at mitigating the systemic impacts of this and similar diseases.

## 2. The Relationship between Skin Diseases and Systemic Inflammatory Diseases

The skin serves not only as the body’s primary barrier against environmental insults, but also as a complex immunological organ [17,18]. Immune cells within the skin perform crucial functions that affect peripheral lymphoid organs [19,20]. They process and respond to external stimuli by conveying signals to various internal organs, facilitating the body’s adaptation to external environmental challenges [21,22]. This advanced capability underscores the development of the skin as an immunologically active organ. These immune cells, inherent to the skin, are not confined to this organ; they migrate to various organs and utilize mechanisms like the release of inflammatory cytokines from the skin to communicate and influence systemic conditions [23,24]. This dynamic illustrates the dual role of the skin’s immune cells: they function both locally and systemically as peripheral lymphoid organs.

The impact of skin inflammation extends beyond the skin itself, affecting multiple other organs and contributing to the pathogenesis of a wide range of systemic inflammatory diseases [25,26,27,28]. Inflammatory conditions such as psoriasis exemplify this phenomenon. Psoriasis, particularly, is associated with a variety of systemic effects driven by inflammatory cytokines [29]. These cytokines can precipitate severe health issues, including cardiovascular events, non-alcoholic fatty liver disease (NAFLD), renal impairment, mental health disorders, and Alzheimer’s disease [30,31,32,33,34]. Clinical evidence suggests that effectively managing and treating psoriasis can significantly mitigate the risk of these associated systemic conditions.

Moreover, other chronic inflammatory skin conditions, such as atopic dermatitis and hidradenitis suppurativa, demonstrate a similar pattern [4,5,35,36,37,38,39,40,41]. Persistent inflammation in these diseases is known to provoke inflammation in organs throughout the body, thereby elevating the overall disease burden and increasing the risk of developing secondary systemic disorders. This relationship highlights the extensive influence that skin-derived inflammation can exert across the entire organism.

Understanding the extensive impact of skin inflammation on systemic diseases illuminates the importance of comprehensive management strategies in dermatological practice. By addressing the inflammatory processes in the skin, clinicians can potentially influence the course of various systemic diseases, offering broader therapeutic options and improving patient outcomes. This integrated approach emphasizes the skin’s role not only as a barrier, but also as a central player in systemic inflammatory pathology, making the management of skin diseases pivotal for dermatological health as well as for overall systemic diseases.

## 3. The Pathogenesis of GPP

**Figure 1:**
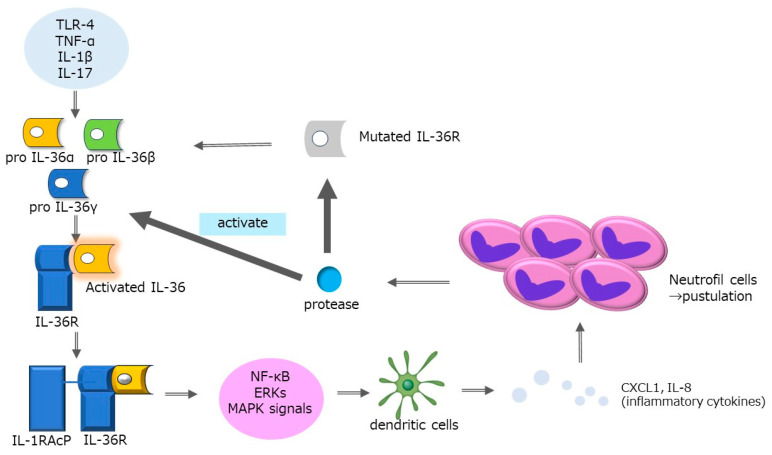
The pathogenesis of GPP. The interleukin-36 (IL-36) pathway is key in generalized pustular psoriasis (GPP), marked by genetic mutations and high levels of IL-36 proteins in GPP lesions. GPP triggers “autoinflammatory” responses through changes in the innate immune system. Neutrophils, a core part of this system, contribute to inflammation by causing oxidative stress, releasing enzymes, and forming neutrophil extracellular traps (NETs). IL-36 cytokines, belonging to the IL-1 family, are uniquely activated outside of cells by proteases from neutrophils and keratinocytes. This activation is critical for starting other inflammation-related pathways involving MYD88, NF-kB, and MAPK. The process is tightly regulated by inhibitors like SERPINA1 and SERPINA3, highlighting the complex control of inflammation in GPP.

The interleukin-36 (IL-36) pathway plays a critical role in GPP [42,43], as evidenced by loss-of-function mutations in the IL36RN gene and related genes such as CARD14, AP1S3, SERPINA3, and MPO, and the overexpression of IL-36 cytokines in GPP lesions [44,45,46,47] (Figure 1). Consistently, clinical improvements have been observed with spesolimab, an anti-IL-36 receptor antibody, indicating a therapeutic potential for targeting this pathway [48]. The pathogenesis of GPP is complex and likely involves both genetic and environmental factors. Significant ongoing research efforts are being made to understand the mechanisms underlying both non-pustular and pustular psoriasis, with some mechanistic overlaps, particularly in patients presenting with both GPP and psoriasis vulgaris (PV) [49,50]. GPP can present independently with unique clinical and histological features, differing in age of onset and sex distribution compared to PV, suggesting distinct underlying mechanisms.

The immunogenetic nature of psoriasis involves an interplay between the innate and adaptive immune systems. In PV, T-cell-derived cytokines like IL-17 are therapeutically targeted with high efficacy [51,52,53,54]. In contrast, GPP involves “autoinflammatory” responses triggered by innate immune alterations [55,56]. Neutrophils, crucial in innate immunity, play a significant role in GPP by contributing to oxidative stress, degranulation, and forming neutrophil extracellular traps (NETs) [57]. The IL-36 cytokines, part of the IL-1 family, are activated extracellularly through proteolytic cleavage by neutrophil- and keratinocyte-derived proteases, unlike the classic IL-1 cytokines that are activated intracellularly [58,59,60]. Activation of IL-36 involves several proteases, with specific cytokines activated by distinct proteases [61,62,63]. The IL-36 signaling pathway is crucial for activating downstream MYD88-mediated pathways such as NF-kB and MAPK [64,65,66]. Negative regulators like SERPINA1 and SERPINA3 modulate this activity, suggesting complex regulation of inflammation in GPP [67,68].

IL-36 cytokines, including IL-36α, IL-36β, and IL-36γ, play crucial roles in regulating immune functions, primarily through the upregulation of several key molecules. These cytokines facilitate robust immune responses by increasing the production of IL-6 and other pro-inflammatory cytokines and chemokines from bone marrow-derived dendritic cells (BMDCs) [69]. They also enhance the expressions of CD80, CD86, and MHC class II on these cells, which improve the ability to present antigens and activate T cells [62].

Furthermore, IL-36γ specifically upregulates ICAM-1 and VCAM-1 on endothelial cells, which aids in monocyte adhesion and promotes chemokine production in human dermal microvascular endothelial cells (HDMEC) [70]. Additionally, IL-36 enhances IFNγ secretion in response to anti-CD3 stimulation in splenocytes and boosts its production in CD4^+^ T cells [62].

In murine studies, IL-36α-treated dendritic cells show increased expressions of CD40, CD80, and CD86, leading to enhanced CD4+ T cell proliferation. These cells also induce Rorc expression and IL-17A production in ovalbumin-specific CD4+ T cells. Moreover, IL-36 enhances the production of IL-1β, IL-6, and IL-23, which promote Th17 differentiation [70].

IL-36 may also exacerbate skin barrier inflammation because treatment of keratinocytes with IL-36γ has been shown to reduce the expression of filaggrin [71]. This action highlights the significant impact of IL-36 on skin structure and function, emphasizing its role in inflammatory processes. Filaggrin is a critical protein for maintaining skin barrier integrity. These findings suggest that IL-36 not only promotes inflammation but also directly affects skin barrier functions in the pathophysiology of inflammatory skin diseases.

Overall, IL-36 cytokines significantly enhance immune and inflammatory responses by upregulating critical cytokines, chemokines, and cellular activation markers, which are essential for effective immune cell function and intercellular communication.

## 4. Systemic Organ Diseases in GPP

This section provides the rationale of various systemic organ diseases related to GPP, such as cardiovascular events, liver dysfunction, renal dysfunction, lung disease, uveitis, anemia, depression, anxiety, and arthritis.

## 5. Cardiovascular Events in GPP

While the connection between psoriasis and the initiation of cardiovascular events is well-established in the medical literature, emerging studies have begun to explore the reciprocal relationship, particularly how cardiovascular events might influence the progression and severity of GPP. Notably, comparing baseline comorbidities, a significant disparity was found between individuals diagnosed with GPP and those with severe psoriasis [72]. Using the French national database (SNDS), three psoriasis cohorts were identified: a prevalent cohort from 2010 to 2018, an incident cohort from 2012 to 2015, and a severe psoriasis cohort. The severe psoriasis cohort was compared with the GPP incident cohort using propensity score matching [72]. For instance, ischemic heart disease was considerably more common in the GPP incident cohort, appearing in 26% of cases, as opposed to in 18% for those with severe psoriasis. This suggests a unique cardiovascular risk profile in GPP patients [72].

Moreover, existing cardiovascular diseases have emerged as critical factors in predicting the clinical outcomes of GPP. A nationwide, population-based retrospective cohort study was conducted to assess adult inpatients with GPP from January 2007 to December 2020 [73]. Research indicates that patients with a history of myocardial infarction are significantly more likely to develop GPP, with an odds ratio of 4.29 after adjusting for age and sex [73]. This correlation becomes even more pronounced in fatal cases of GPP, where the history of myocardial infarction carries an odds ratio of 5.10 after adjusting for age and sex, highlighting a strong link between severe cardiovascular conditions and lethal outcomes in GPP [73].

Despite these findings, the impact of GPP on the occurrence of cerebrovascular diseases remains poorly defined, with few studies directly addressing this aspect. This gap in understanding points to the need for further research into how GPP may affect or be affected by cerebrovascular conditions.

Lastly, the biological mechanisms through which GPP may regulate or be influenced by cardiovascular events are still not fully understood. This area of study is crucial, as unraveling these mechanisms could lead to better targeted therapies and preventive strategies for patients suffering from both GPP and cardiovascular diseases.

Interleukin-36 (IL-36) is increasingly recognized as a pivotal trigger in the production of interleukin-17A (IL-17A) within the skin [70,74,75], which in turn plays a crucial role in the development of atherosclerosis, a major contributor to cardiovascular disease [76,77]. This pathway underscores a significant link between inflammatory processes in dermatological conditions and broader vascular health issues. The mechanism through which IL-36 prompts the release of IL-17A involves a complex cascade of immune responses, which ultimately lead to inflammation and subsequent changes in the vascular walls, facilitating atherosclerotic developments.

In light of this, therapies targeting IL-17A have garnered attention for their potential to not only manage psoriasis, but also to mitigate associated vascular inflammation that can lead to serious cardiovascular events. One such therapy, secukinumab, which functions as an IL-17A inhibitor, has shown promising results in reducing vascular inflammatory markers in patients with psoriasis [78,79]. This effect suggests that IL-17A blockage could serve as a strategic intervention to decrease the future risk of cardiovascular events in these patients.

The implications of such treatments are profound, as they offer a dual benefit: alleviating the skin symptoms of psoriasis, while simultaneously addressing the heightened risk of cardiovascular diseases linked to systemic inflammation. Ongoing research and clinical trials are crucial to further elucidate the efficacy and safety of IL-17A inhibitors, potentially paving the way for novel therapeutic strategies that integrate the management of psoriasis and cardiovascular risk. This could fundamentally alter the therapeutic landscape, providing a more holistic approach to treating patients with psoriasis by not only improving skin health, but also enhancing cardiovascular outcomes.

While the specific risk of cerebrovascular diseases associated with GPP has not been extensively documented, insights can be drawn from the underlying mechanisms of inflammation common to both GPP and other forms of psoriasis, particularly psoriasis vulgaris. In psoriasis vulgaris, the link between inflammation and cerebrovascular events is increasingly being recognized, largely due to the role of cytokines such as interleukin-17 (IL-17) and interleukin-36 (IL-36).

IL-36, a cytokine prominently featured in the inflammatory pathways of GPP, acts as a potent inducer of IL-17. This induction is critical because IL-17 has been implicated in the progression of atherosclerosis—a key factor in the development of cerebrovascular diseases. The inflammation mediated by IL-17 in this context not only contributes to the structural changes within the vascular walls, but also exacerbates the instability of these plaques, increasing the risk of cerebrovascular accidents such as strokes.

IL-36 and its receptor IL-36R were detected in the hearts of mice experiencing ischemia/reperfusion (IR) injury [80]. Treatment with an IL-36 receptor antagonist reduced neutrophil recruitment and improved coronary circulation [80], suggesting that the protective effects of targeting IL-36 may stem from decreased oxidative damage to endothelial cells and reduced expression of VCAM-1 in cardiovascular events.

IL-36γ also plays a key role in modulating inflammatory processes and lipid metabolism in macrophages, which facilitates atherosclerosis through increased formation of macrophage foam cells and enhanced uptake of oxidized low-density lipoproteins [81]. Specifically, IL-36γ upregulates the scavenger receptor CD36 through the activation of the phosphoinositide 3-kinase pathway in macrophages [81]. This research highlights IL-36γ as an emerging contributor to foam cell development and the progression of atherosclerosis.

Given the similarity in inflammatory pathways between GPP and psoriasis vulgaris, it is plausible to hypothesize that GPP, through the same mechanisms involving IL-17 and IL-36, might similarly enhance the risk of cerebrovascular events. This potential risk underscores the importance of further research into the inflammatory profiles of patients with GPP, with a specific focus on their cardiovascular and cerebrovascular events. Understanding these connections in greater depth could lead to more effective strategies for monitoring and managing the risks of cerebrovascular diseases in patients suffering from GPP, potentially aligning treatment approaches with those used in managing psoriasis vulgaris where the risk is better understood.

## 6. Liver Dysfunction in GPP

Some isolated cases of GPP have indicated its possible involvement with liver disorders. A previous study examined the prevalence and characteristics of liver abnormalities in GPP. Twenty-two patients diagnosed with GPP underwent liver function tests, during an acute episode and in subsequent weeks [82]. This study focused on patients consecutively admitted for a GPP attack at one of two dermatology departments, where the diagnosis of GPP was confirmed based on clinical criteria validated by two dermatologists and histological analysis of skin biopsies. Remarkably, 90% (20 patients) displayed at least one abnormal liver examination result. Half of the patients exhibited significant liver disturbances: jaundice was observed in four cases (18%), elevated gamma-glutamyl transferase (more than five times the normal limit) in ten cases (45%), high alkaline phosphatase (over double the normal limit) in seven cases (32%), and increased aminotransferases (more than three times the normal limit) also in seven cases (32%). These liver abnormalities normalized upon the remission of the psoriasis, indicating a potential link between severe liver issues and the intensity of the skin manifestations.

The specific biological pathways through which GPP leads to liver impairment are not fully understood. However, there is a particularly notable case that sheds light on this issue. In this instance, a patient suffering from primary biliary sclerosis (PBS), which was exacerbated by GPP, experienced significant improvements in both skin inflammation and liver function following treatment with an IL-17 antibody [83]. Furthermore, detailed histological analysis revealed the presence of IL-17-producing cells within the liver tissue, specifically concentrated in the portal tract areas [83]. This observation supports the hypothesis that GPP may induce liver damage through a mechanism involving the activation of IL-17 by IL-36. This finding not only enhances our understanding of the inflammatory mechanisms at play, but also suggests that therapies targeting IL-17 could have a dual benefit. They might not only alleviate the cutaneous symptoms of GPP, but also offer protective effects against liver damage. This dual potential makes the role of IL-17 inhibitors a promising area for further research in the treatment strategies for GPP, especially for those patients presenting with concurrent liver complications.

Although the specific mechanisms of liver diseases such as non-alcoholic fatty liver disease (NAFLD), commonly associated with psoriasis, have not been thoroughly delineated in the context of GPP, it is plausible to consider that similar pathophysiological processes could potentially be involved the development of liver dysfunction in GPP. In cases of GPP, the release of IL-36, which in turn stimulates the production of IL-17, might contribute to the development of these liver conditions. Given the intricate interplay of these cytokines in inflammatory responses, it is conceivable that the activation of IL-17 by IL-36 in GPP could also trigger or exacerbate liver diseases such as NAFLD [84,85,86,87]. This connection warrants a deeper exploration to understand the full spectrum of systemic implications associated with GPP. Future research in this area is not only crucial but also expected to provide significant insights that could lead to better management and treatment strategies for patients suffering from both GPP and associated liver diseases.

## 7. Renal Dysfunction in GPP

A previous study conducted a literature review to identify research on mortality, prevalence, comorbidities, and specific mutations in the GPP population, executing the search in Embase, Medline, and the Cochrane Library in April 2021 and supplementing it with a review of relevant literature and consensus studies identified in these databases until October 2021, alongside a search for congress abstracts [14]. GPP increased the risk of renal disease (odds ratio, 7.31) [14]. Cohort studies have increasingly highlighted that patients with pustular psoriasis face a heightened risk of renal dysfunction, suggesting a notable vulnerability within this population. Furthermore, case reports have further underscored this connection, documenting instances where GPP was associated with the onset of renal failure, thereby implicating GPP in potentially severe renal complications [88]. Although the precise pathological mechanisms behind this association remain elusive, it is theorized that the inflammatory processes inherent to GPP, particularly through the action of cytokines such as IL-17, may play a critical role [89,90,91]. IL-17 is typically produced following the activation of IL-36, a cytokine that is significantly involved in inflammatory responses to cause kidney fibrosis in GPP [92]. A previous study has shed light on the role of IL-36 beyond its immediate inflammatory effects, particularly its contribution to fibrotic processes. Given that fibrosis can be a major factor in renal dysfunction, the involvement of IL-36 in promoting renal fibrosis presents a plausible pathway through which GPP could exacerbate or directly cause renal impairment [93,94]. This connection between IL-36-induced cytokine activity and renal fibrosis offers a promising area for further scientific inquiry.

Understanding these complex interactions in greater depth will be crucial for developing targeted therapies that can mitigate the renal risks associated with GPP. As such, the medical community eagerly anticipates future studies that will clarify these mechanisms, potentially leading to more effective treatments for patients suffering from GPP and its systemic manifestations.

## 8. Lung Disease in GPP

A previous cohort study utilized data from the Medical Data Vision claims database in Japan that included 718 patients with GPP and 27,773 with plaque psoriasis; focusing on the prevalence of comorbidities, medication use, and healthcare resource utilization, the results showed a higher incidence of interstitial pneumonia [95]. Although the direct relationship between GPP-related IL-36 and lung disease remains unclear, another study examined the role of IL-36 cytokines in rheumatoid arthritis related interstitial lung disease (RA-ILD) [96]. It found that plasma concentrations of IL-36α and IL-36γ were higher in RA-ILD patients compared to healthy controls and RA patients without ILD [96], suggesting a possible role of IL-36 in lung diseases.

## 9. Osteoporosis in GPP

Results from the Medical Data Vision claims database in Japan show that GPP cases exhibit a higher incidence of osteoporosis compared to those afflicted with the more common plaque type of psoriasis [95,97]. Although direct connections between IL-36 and osteoporosis have not been explicitly documented in the literature, the link is well-established between increased osteoporosis risk and psoriasis, particularly plaque psoriasis. This relationship suggests a potential underlying inflammatory mechanism common to both conditions. Investigation into the role of cytokines reveals that IL-17, which is induced by IL-36, plays a significant role in the pathogenesis of osteoporosis. Research involving osteoporosis models, particularly in mice that have undergone ovariectomy—a procedure that simulates post-menopausal osteoporosis in humans—demonstrates that antibodies neutralizing IL-17 can effectively slow down or inhibit the progression of bone loss [98]. This is particularly relevant because IL-17 is known to contribute to the activation of osteoclasts, the cells primarily responsible for bone resorption.

Given these findings, it is plausible to infer that the inflammatory processes associated with GPP, mediated by IL-36 and IL-17, may extend beyond mere skin inflammation to significantly contribute to the higher prevalence of osteoporosis observed in these patients. This connection underscores the importance of considering systemic inflammatory markers like IL-36 and IL-17, not only as potential targets for treating skin symptoms, but also for addressing associated systemic complications such as osteoporosis in patients with GPP. Such an approach could lead to more comprehensive treatment strategies that address both the dermatological and skeletal aspects of the disease.

## 10. Uveitis in GPP

A previous case report showed a rarely exhibited uveitis in a patient with GPP [99]. Currently, the connection between GPP and the risk of developing uveitis remains insufficiently explored and lacks clear analytical evidence. Despite this gap in research, there are intriguing indications regarding the potential involvement of specific cytokines in the pathology of uveitis, which may be relevant to patients with GPP.

Although no studies have directly established a causal link between IL-36 and the development of uveitis, emerging research points towards an association involving other related cytokines, particularly IL-17. Observations in clinical settings have shown that patients with uveitis frequently exhibit elevated levels of IL-17 [100]. This correlation is further supported by experimental studies on animal models, where an increase in IL-17 has consistently been noted in cases of uveitis [101,102].

Moreover, interventions targeting IL-23, a cytokine upstream of IL-17, have demonstrated promising results in these models. By inhibiting IL-23, researchers have been able to not only decrease the levels of IL-17, but also significantly reduce the histological signs of inflammation associated with uveitis [102]. These findings suggest a complex interplay between IL-17 and IL-23 in driving the inflammatory processes underlying uveitis, hinting at potential therapeutic targets that might also be applicable in the context of GPP-associated uveitis.

This speculative link between GPP, IL-17, and uveitis highlights the need for more comprehensive studies to better understand the immunological mechanisms at play, and to evaluate the effectiveness of cytokine-targeted therapies in managing uveitis in patients with GPP.

## 11. Anemia in GPP

Anemia is a notable and common complication associated with GPP. A retrospective cohort analysis was conducted on GPP patients hospitalized between January 2010 and November 2022 to elucidate the influence of GPP in anemia [103]. In a comparative study involving 416 patients, each diagnosed with GPP and psoriasis vulgaris, anemia was found to be significantly more prevalent in patients with GPP than in those with PV (13.9% vs. 1.2%, *p* < 0.001) [103]. Additionally, a separate cohort study of 860 GPP patients highlighted the impact of severe GPP on anemia [104]. In order to clarify the association between GPP and anemia, this study utilized the National Inpatient Sample, a publicly available, de-identified database of U.S. hospital encounters produced by the Agency for Healthcare Research and Quality (AHRQ), from the years 2016 to 2020. This cohort demonstrated that the mean hospital stay for patients with anemia was significantly longer at 9.6 days, compared to 5.2 days for those without anemia. Furthermore, the cost of care was substantially higher for the anemia group, averaging USD 14,919, as opposed to USD 9862 for the non-anemia group. Both the length of stay and cost of care showed significant differences between the two groups in univariate analysis (*p* < 0.001). This underscores the importance of thorough evaluation and treatment of anemia in patients with GPP as critical components of their clinical management.

## 12. Depression and Anxiety in GPP

Patients with GPP experience a significant psychological disease burden, exacerbated by the physical and psychological challenges of the disease. Depression and anxiety are prevalent among GPP sufferers due to chronic pain, fatigue, and the visible nature of the disease. A retrospective cohort study was performed on GPP patients, admitted between January 2010 and November 2022, to investigate the association with psychological disorders [105]. Descriptively, patients with generalized pustular psoriasis (GPP) not only reported higher levels of anxiety and depression compared to those with other forms (38% vs. 26%), but they also had more extensive treatment histories, with a higher percentage having undergone at least two previous systemic treatments (15% vs. 7%) [105]. The direct influence of IL-36 on depression or anxiety has not been clearly established. However, a mouse model subjected to chronic, unpredictable, mild stress demonstrated depressive behavior linked to an upregulation of pro-inflammatory T helper 17 (Th17) cells in the liver and ileum [106]. This animal study’s results also showed increased levels of interleukin IL-17; treatment targeting IL-17 alleviated symptoms of anxiety and depression [107]. These findings suggest that systemic treatment for psoriasis could potentially mitigate the risk of psychological disorders.

## 13. Arthritis in GPP

A significant proportion of GPP patients, approximately 20%, develop psoriatic arthritis (PsA), suggesting a deep interconnection between cutaneous and joint manifestations in psoriasis [108]. The notable rapid progression from the onset of psoriatic arthritis to the diagnosis of GPP highlights the critical need for understanding the underlying mechanisms that link these conditions [109].

A retrospective cohort study aimed to identify factors associated with GPP diagnosis among psoriasis patients selected from the Japanese Medical Data Center database, spanning 1 July 2005 to 31 January 2019 [109]. The strong association between GPP and PsA is evident, with the odds ratio of developing GPP being significantly higher in patients with PsA [109]. The median time of 119 days from the event of psoriatic arthritis to GPP diagnosis underscores the aggressive nature of this linkage and prompts further investigation into the inflammatory pathways involved.

IL-36 plays a pivotal role in promoting inflammation, and has been extensively studied in the context of psoriasis and PsA [110]. Synovial tissue analyses from PsA and rheumatoid arthritis (RA) patients reveal that IL-36 levels are distinctly higher in PsA, with a notable imbalance in IL-36 antagonists and agonists. This imbalance contributes to the sustained inflammation observed in PsA, even post-DMARDs therapy, indicating a potentially unique inflammatory pathway in PsA compared to RA. Understanding the precise role of IL-36 in these inflammatory processes could lead to more effective treatment strategies that address both the dermatological and arthritic components of the disease.

## 14. Conclusions

**Figure 2:**
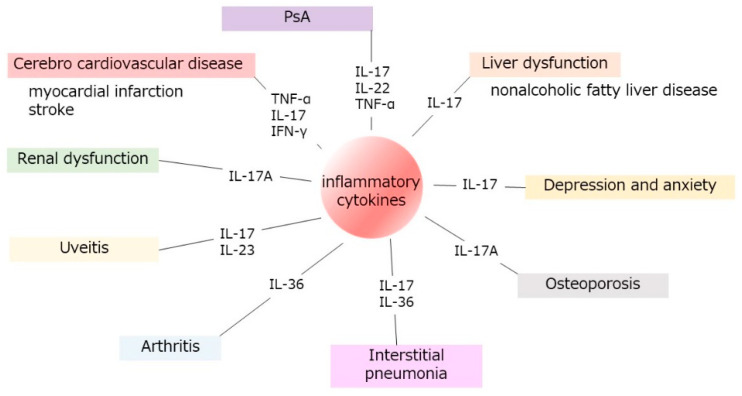
The interplay of GPP and systemic organ dysfunctions. This schema displays the connections between generalized pustular psoriasis (GPP) and various systemic diseases, showing how GPP significantly affects the cardiovascular, liver, renal, pulmonary, and skeletal systems, as well as depression, anxiety, and arthritis. GPP-derived IL-36 and associated IL-17 cytokines show interplay in the development of systemic organ dysfunctions.

This review highlights the complex interplay between GPP and various systemic diseases, emphasizing its significant impact on cardiovascular, liver, renal, pulmonary, and skeletal systems (Figure 2) (Table 1). In terms of cardiovascular risk, GPP is associated with an increased incidence of cardiovascular events, such as ischemic heart disease, which appear at higher rates compared to those with severe psoriasis. Existing research also shows a strong correlation between a history of myocardial infarction and the likelihood of developing GPP, suggesting profound cardiovascular implications for these patients.

Regarding liver dysfunction, this review notes a high prevalence of liver abnormalities in patients with GPP, with these disturbances often normalizing upon remission of the skin disease. This observation suggests a potential link between GPP and liver health, possibly mediated by cytokines like IL-17, which may contribute to liver damage.

The research also points to an elevated risk of renal dysfunction in GPP patients, with cytokines such as IL-17 and IL-36 playing a role in the inflammatory process that may lead to renal fibrosis and dysfunction. Additionally, the increased incidence of interstitial lung disease (ILD) in patients with rheumatoid arthritis mirrors similar pulmonary conditions in GPP, where IL-36 and IL-17 may exacerbate pulmonary issues.

Furthermore, GPP patients show a higher incidence of osteoporosis, likely due to inflammatory mechanisms involving IL-36 and IL-17 that influence bone resorption. Overall, this comprehensive examination underscores the need for a holistic approach to managing GPP, considering its extensive systemic effects beyond the skin. Continued research into the mechanisms linking GPP with these systemic conditions is crucial for developing targeted therapies that address both the dermatological symptoms and associated systemic complications.

## Figures and Tables

**Table 1:** The collected epidemiological data on comorbidities in GPP.

The Comorbidities	The Risks or Frequency in GPP
Cardiovascular events	Increased frequency [72]
Liver disturbances	Increased frequency [82]
Renal dysfunction	Increased risk [14]
Interstitial pneumonia	Increased frequency [95]
Osteoporosis	Increased frequency [95,97]
Anemia	Increased frequency [103]
Depression and anxiety	Increased frequency [105]
Arthritis	Increased frequency [108,109]
